# Prevalence of ophthalmic abnormalities and refractive changes in Taiwanese children with Down syndrome

**DOI:** 10.1371/journal.pone.0324366

**Published:** 2025-05-21

**Authors:** Chia-Ping Chen, Tzu-Hsun Tsai, Yao-Lin Liu, Ni-Chung Lee, Che-Ning Yang, Chao-Wen Lin

**Affiliations:** 1 Department of Medical Education, National Taiwan University Hospital, Taipei, Taiwan; 2 Department of Ophthalmology, National Taiwan University Hospital, Taipei, Taiwan; 3 Department of Ophthalmology, National Taiwan University Hospital, Hsin-Chu Branch, Hsin-Chu, Taiwan; 4 Department of Medical Genetics, National Taiwan University Hospital, Taipei, Taiwan; 5 Department of Pediatrics, National Taiwan University Hospital, Taipei, Taiwan; 6 School of Medicine, National Taiwan University, Taipei, Taiwan.; Alexandria University Faculty of Medicine, EGYPT

## Abstract

**Background:**

Ophthalmological manifestations in children with Down syndrome remain significantly underdiagnosed, particularly in Asian populations. This study aimed to investigate the prevalence and characteristics of ophthalmic disorders in Taiwanese children with Down syndrome, analyze their refractive changes over time, and compare findings with existing research across diverse ethnic populations.

**Methods:**

Comprehensive ophthalmologic examinations were conducted on 148 Taiwanese children with Down syndrome, documenting age, sex, medical history, ocular alignment, visual acuity, and cycloplegic refraction. Annual refractive change assessments were performed using multivariate linear regression analysis for subjects with minimum one-year follow-up.

**Results:**

Visually significant refractive errors were identified in 75.7% of subjects, with astigmatism being most prevalent (70.3%), followed by hyperopia (31.8%) and myopia (19.6%). Our cohort showed higher astigmatism prevalence compared to previous studies of Asian children with Down syndrome. Longitudinal analysis revealed an annual refractive change of −0.16 ± 0.65 diopters, with age and baseline spherical equivalence emerging as significant contributing factors.

**Conclusions:**

This study provides unprecedented data on ophthalmic disorders and refractive changes in Taiwanese children with Down syndrome. The high prevalence of refractive errors, particularly astigmatism, underscores the importance of early and regular screening in this population. These findings may inform the development of targeted screening protocols and optimal intervention timing.

## 1. Introduction

Down syndrome (DS) is a significant chromosomal disorder affecting approximately 1 in 1,000 live births globally [1], though Taiwan reports a notably lower incidence of 7.9 per 10,000 live births annually due to comprehensive prenatal screening programs [2]. This genetic condition, caused by meiotic nondisjunction resulting in an additional copy of chromosome 21, manifests through extensive multisystemic complications including neurological, musculoskeletal, and cardiovascular abnormalities [3].

Visual problems represent a particularly significant concern, affecting 60% to 80% of individuals with DS [4]. The spectrum of ophthalmic manifestations encompasses eyelid abnormalities, refractive errors, strabismus, nystagmus, cataract, amblyopia, nasolacrimal duct obstruction, glaucoma, and keratoconus [2,5–7], as previous research focused on DS individuals from Asia, Africa, Americas and Europe. Individuals with DS exhibited a markedly higher prevalence of refractive errors compared to the general population, while whether hyperopia [8–11] or myopia [5,12] as the predominant refractive error was still in debate. In non-selected DS children, the prevalence of strabismus varied widely from 5.6% to 65.0% [11,13]. Esotropia was the most common type among strabismus in children with DS and usually occurred around age three to six years old, which was older than the time of acquired esotropia developed in typically developing children around the age of two [14,15]. A combination of factors, including reduced fusional capacity, diminished visual resolution capacity, and an insufficient accommodative convergence may contribute to the development of strabismus in children with DS [7].

However, some ocular disorders, such as cataracts, refractive errors and keratoconus, typically manifest during later childhood or adolescence, while early identification and correction of these conditions, can substantially enhance visual function and quality of life. Therefore, the significance of systematic monitoring is underscored by the temporal patterns of certain ocular conditions in DS children.

Amblyopia meets the World Health Organization’s criteria for a condition that benefits from screening. It is a significant health concern with an established treatment, and reliable diagnostic methods are available to detect the condition before permanent vision loss occurs. According to the American Academy of Ophthalmology (AAO) Preferred Practice Patterns [16], early detection and intervention are essential during critical periods of visual development, with screening approaches tailored to each child’s developmental stage. For infants, evaluation consists of red reflex test, fixation and following assessment, and external inspection. As children reach ages three to four years, standardized visual acuity testing with validated optotypes such as Snellen chart, Tumbling E chart, LEA symbols becomes appropriate. However, children with DS frequently experience developmental delays that limit cooperation with traditional visual acuity testing methods. For these children, instrument-based screening technologies, including photoscreening, retinoscopy, and autorefraction, are particularly valuable for detecting significant refractive errors during early childhood when conventional testing remains challenging. These objective assessment methods enable clinicians to identify visually significant refractive errors requiring correction, thereby potentially reducing amblyopia occurrence in this vulnerable population who demonstrate higher prevalence of vision abnormalities. The timely prescription of appropriate optical correction based on these evaluations represents a critical intervention for optimizing visual outcomes in children with DS.

Our study investigated and analyzed the prevalence of ophthalmic abnormalities among Taiwanese children with DS under 18 years of age. The research objectives are twofold: first, to conduct a comparative analysis of our findings against published studies examining DS children across various ethnicities and countries; and second, to evaluate refractive changes in DS children longitudinally during the follow-up period. We aimed to calculate the annual refractive progression and paid particular attention to the influence of both demographic and ophthalmological factors on these changes.

## 2. Materials and methods

### 2.1. Subjects and study design

Subjects with DS who were younger than 18 years at the time of their first ophthalmological examination at the special clinic between 2014 and 2022 were included in the study. Individuals were excluded if they were older than 18 years or had incomplete ophthalmological examination records. All ophthalmic examinations were carried out in a dedicated clinical environment following established diagnostic protocols. Subjects data, including their age, sex, medical history, ocular motility and alignment assessed using the Hirschberg test or prism cover test, visual acuity assessed using the Tumbling E chart, and cycloplegic refraction status, were collected. For DS children who were unable to demonstrate directional awareness with finger pointing when tested with Tumbling E chart, we opted for LEA symbols chart as substitute. During ocular motility and alignment assessment, we used TV programs to attract their attention and employed objects of interest to the children to maintain their focus.

Cycloplegic refraction involved administering three consecutive drops of 0.5% tropicamide at five-minute intervals. Refraction was then performed using either an autorefractor or retinoscope 30 minutes after the last tropicamide instillation. DS children were guided to focus on the house-configured fixation stimulus presented by the autorefractor through verbal cues provided by our optometrists. For DS children who remained unable to maintain adequate fixation with the autorefractor, we also used TV programs to attract their attention and employed retinoscopy as an alternative method for obtaining accurate refractive measurements. All significant ophthalmic findings were recorded. The study adhered to the tenets of the Declaration of Helsinki and was approved by the Institutional Review Board of National Taiwan University Hospital (202206019RINB). The data were accessed retrospectively from November, 2022 to May, 2023. Although the authors had access to information that could identify individual participants during data collection, we replaced medical record numbers with unique codes and ensured that no identifiable information was included in the recorded data. The mapping table linking the codes to medical record numbers was encrypted and stored separately. The Institutional Review Board approved the use of retrospective medical records without obtaining informed consent.

To assess the prevalence of ophthalmic abnormalities at various developmental stages, these DS children were classified into three age subgroups: younger or equal to six years old, over six to 12 years old, and over 12–18 years old, expressed as early childhood, middle childhood and adolescence. For the analysis of annual refractive change, we considered children with DS with complete refraction data who had undergone regular follow-up for at least one year after their initial visit. The changes in refractive errors were expressed as spherical equivalence in diopters (D). Annual refractive changes were calculated by dividing the change in spherical equivalence by the number of years of follow-up.

### 2.2. Definition of refractive errors and visually significant refractive errors

Myopia was defined as spherical equivalence of −0.5 D or less. Hyperopia was defined as spherical equivalence of 0.5 D or more. On the other hand, the definition of visually significant refractive errors varied across age groups (S1 Table). For children younger than four years, we referred to the *Pediatric Eye Evaluations Preferred Practice Pattern* published by the AAO in 2018 [16]. For children older than four years old, we applied the uniform definitions from 2013 version of American Association for Pediatric Ophthalmology and Strabismus (AAPOS) *Vision Screening Committee Guidelines* [17]. The guideline established age-stratified thresholds across five developmental categories: younger than one year, one to two years, two to three years, three to four years, and older than four years. Threshold values demonstrate an inverse relationship with advancing age: myopia thresholds range from > 5.0 D in infants to > 1.5 D in children exceeding four years of age; hyperopia without deviation ranges from > 6.0 D to > 3.5 D; hyperopia with esotropia ranges from > 2.0 D to > 1.5 D; and astigmatism ranges from > 3.0 D to > 1.5 D in cylindrical power. For anisometropia, distinct thresholds were applied for myopic, hyperopic, and astigmatic interocular differences, all following a similar age-dependent progression with diminishing thresholds correlating with increasing age.

### 2.3. Statistical analysis

The results are expressed as means ± standard deviations. To compare the prevalence of ophthalmic findings in various age subgroups, the Fisher–Freeman–Halton exact test was employed. One-way analysis of variance with a Bonferroni post hoc test was employed to compare the annual refractive changes across age subgroups. A multivariate linear regression model was used to investigate the factors associated with refractive changes. *P* values less than 0.05 were considered significant. STATA, version 14 (Stata, College Station, TX, USA), was used for all statistical analyses.

## 3. Results

### 3.1. Prevalence of ophthalmic abnormalities in children with DS

**Fig 1:**
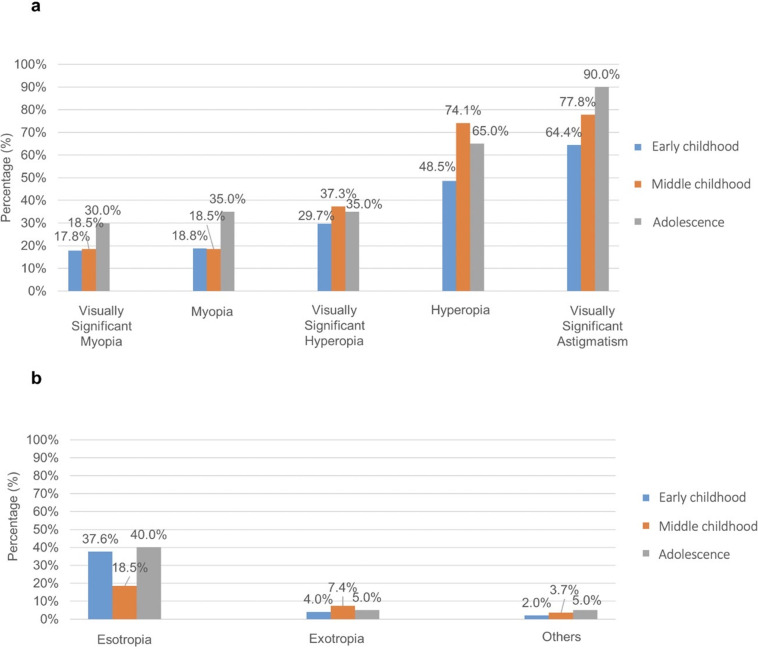
Prevalence of refractive errors, visually significant refractive errors (a), and strabismus types (b) in children with Down syndrome (DS).

A total of 148 children (82 boys and 66 girls) were included in the study, with a mean age at their first visit of 64.9 ± 56.2 months. The subgroups of early childhood, middle childhood and adolescence comprised 101, 27, and 20 children, respectively. Ophthalmic abnormalities were observed in 132 children (89.2%). Refractive errors were the most common findings, with 31 children (20.9%) having myopia (spherical equivalence ≤ −0.5 D), and 82 (55.4%) having hyperopia (spherical equivalence ≥ 0.5 D). In addition, 112 children (75.7%) were given a diagnosis of visually significant refractive errors according to the *Pediatric Eye Evaluations Preferred Practice Pattern published* by AAO and *Vision Screening Committee Guidelines* published by AAPOS (S1 Table), including visually significant myopia (29, 19.6%), visually significant hyperopia (47, 31.8%), visually significant astigmatism (104, 70.3%), and visually significant anisometropia (12, 8.1%) (Fig 1a). A total of 62 children (41.9%) exhibited strabismus, with 51 having esotropia, seven having exotropia, three having inferior oblique muscle overaction, and one having dissociated vertical deviation (Fig 1b). Nystagmus was observed in 41 children (27.7%), nasolacrimal duct obstruction in 12 children (8.1%), and glaucoma in four children (2.7%). Cataracts were found in 15 children (10.1%), with a predominance in adolescents. No children were given a diagnosis of keratoconus. Only cataracts show a statistically significant difference across age subgroups, while all other conditions demonstrate no statistically significant differences in prevalence across the age subgroups. The detailed data are presented in Table 1.

**Table 1:** Ophthalmic abnormality prevalence in children with Down syndrome.

Age groups	Early childhood	Middle childhood	Adolescence	P value	Total
Number of children	101	27	20		148
Mean age of first visit (months)	32.8	99.8	179.5		64.9 ± 56.2
Visually significant refractive error	71 (70.3%)	22 (81.5%)	19 (95.0%)	0.526	112 (75.7%)
Myopia	18 (17.8%)	5 (18.5%)	6 (30.0%)	0.475	29 (19.6%)
Hyperopia	30 (29.7%)	10 (37.3%)	7 (35.0%)	0.206	47 (31.8%)
Astigmatism	65 (64.4%)	21 (77.8%)	18 (90.0%)	0.598	104 (70.3%)
Anisometropia	6 (5.9%)	4 (14.8%)	2 (10.0%)	0.666	12 (8.1%)
Strabismus	44 (43.6%)	8 (29.6%)	10 (50.0%)	0.493	62 (41.9%)
Esotropia	38 (37.6%)	5 (18.5%)	8 (40.0%)	0.708	51 (34.5%)
Exotropia	4 (4.0%)	2 (7.4%)	1 (5.0%)	0.229	7 (4.7%)
Others	2 (2.0%)	1 (3.7%)	1 (5.0%)		4 (2.7%)
Nystagmus	29 (28.7%)	7 (25.9%)	5 (25.0%)	0.059	41 (27.7%)
Cataract	4 (4.0%)	4 (14.8%)	7 (35.0%)	**<0.001**	15 (10.1%)
Nasolacrimal duct obstruction	10 (9.9%)	2 (7.4%)	0 (0.0%)	0.639	12 (8.1%)
Glaucoma	3 (2.97%)	0 (0.0%)	1 (5.0%)	0.437	4 (2.7%)
Keratoconus	0 (0.0%)	0 (0.0%)	0 (0.0%)	1.000	0 (0.0%)

### 3.2. Annual refractive changes in DS children

In the examinations of the 148 children, 134 eyes from 67 children (35 boys and 32 girls) had complete records of refractive status with a follow-up period of more than one year, with an average follow-up period of 4.06 ± 2.47 years. The follow-up periods were 3.77 ± 2.18 years, 4.19 ± 2.66 years, and 4.79 ± 2.95 years for the age subgroups of early childhood, middle childhood and adolescence, respectively. Statistical analysis revealed no significant differences between the left and right eyes. The groups of children in early childhood, middle childhood and adolescence with complete records comprised 38, 17, and 12 children, respectively. For refractive change analysis, we excluded eyes with cataracts due to potential confounding. Of the remaining 116 eyes, 41 myopic eyes (spherical equivalence ≤ −0.5 D) and 56 hyperopic eyes (spherical equivalence ≥ 0.5 D) were included in subgroup analysis. In all children with complete refraction data, the annual refractive changes were −0.16 ± 0.65 D, 0.02 ± 0.67 D, and −0.29 ± 0.62 D in the total, myopic, and hyperopic eye subgroups, respectively. In the hyperopic subgroup, the adolescents exhibited a significantly higher annual refractive change compared with the subgroups of children in early childhood (*P* = 0.0371) and middle childhood (*P* = 0.0292) by Bonferroni post hoc test. The detailed data are presented in Table 2.

**Table 2:** Annual refractive changes in children with Down syndrome in various age subgroups.

Groups	Subgroups	Annual refractive changes ± standard deviation	ANOVA (*P* value)
All	Early childhood	-0.13 ± 0.69D	0.3722
	Middle childhood	-0.12 ± 0.38D	
	Adolescence	-0.34 ± 0.83D	
	Overall	-0.16 ± 0.65D	
Myopia	Early childhood	0.03 ± 0.83D	0.9617
	Middle childhood	-0.03 ± 0.47D	
	Adolescence	0.02 ± 0.46D	
	Overall	0.02 ± 0.67D	
Hyperopia	Early childhood	-0.25 ± 0.54D	**0.0264**
	Middle childhood	-0.15 ± 0.32D	
	Adolescence	-0.91 ± 1.22D	
	Overall	-0.29 ± 0.62D	

### 3.3. Associated factors of refractive changes in DS children

Linear regression analysis revealed that age and baseline spherical equivalence substantially influenced refractive changes, and indicated that older DS children and DS children with higher spherical equivalence exhibited a greater tendency for myopic shift. In the myopic eyes subgroup, visually significant astigmatism was identified as the only significant factor influencing myopic progression. In the hyperopic eyes subgroup, factors including male sex, age, esotropia, and higher baseline spherical equivalence were identified as contributing to myopic shift. The detailed data are presented in Table 3.

**Table 3:** Factors associated with refractive changes in children with Down syndrome.

	All		Myopia		Hyperopia	
	Coefficient (Standard error)	P value	Coefficient (Standard error)	P value	Coefficient (Standard error)	P value
Sex Male = 1	-0.071 (0.121)	0.562	0.360 (0.194)	0.072	-0.382 (0.15)	***0.014***
Age	-0.053 (0.014)	***<0.001***	-0.019 (0.024)	0.420	-0.037 (0.017)	***0.038***
Strabismus						
Esotropia	-0.107 (0.127)	0.399	0.405 (0.203)	0.054	-0.546 (0.154)	***0.001***
Baseline spherical equivalence	-0.042 (0.014)	***0.005***	-0.016 (0.022)	0.486	-0.101 (0.044)	***0.026***
Visually significant astigmatism	-0.213 (0.215)	0.324	-1.245 (0.329)	***0.001***	0.122 (0.279)	0.665

## 4. Discussion

This study examines the prevalence of ophthalmological disorders in Taiwanese children with DS at the special clinic of a tertiary medical center. The research makes a unique contribution to the field by providing unprecedented data on the prevalence of ocular abnormalities and annual refractive changes in Taiwanese DS children, while also investigating the demographic and clinical factors associated with these refractive changes across different refractive status subgroups. Our study has substantial clinical and public health implications for ophthalmologic care in children with DS, given that many of these ocular manifestations are amenable to intervention. Implementation of early screening protocols may be crucial for preventing vision loss in this vulnerable population.

In Table 1, cataract prevalence was the only ophthalmic condition demonstrating significant age-related variation in our DS cohort. Although congenital and cerulean cataracts are more common in DS populations and are now more easily detected through newborn red reflex screening, our age-related findings present an intriguing contrast to existing literature. While one study [18] of older DS children over 30 years old found cataracts in 72.2% of eyes (26.1% congenital, 44.9% age-related), yet another study [19] found no evidence supporting early-onset age-related cataracts. Further longitudinal studies are required to better understand the progression of cataract development in children with DS.

To compare with the ophthalmic abnormalities research regarding DS children, we did a comprehensive literature research on this topic. In Table 4 [5,6,9–13,20–33], a comprehensive comparison of ophthalmic abnormality prevalence in DS children across different geographical regions, including our current study in Taiwan. Our study included 148 children with DS, 82 males and 66 females aged 0–18 years, representing one of the larger cohorts among the comparative studies. We systematically categorized studies according to geographic region and ethnicity as Asia, Europe, Americas, and Africa. For each study, we documented the following parameters: country or region, age range of participants, sample size, and prevalence expressed as percentages of various ophthalmic abnormalities in DS children. The documented ophthalmic findings included overall abnormality rates, myopia, hyperopia, astigmatism, and visually significant refractive errors based on age-stratified criteria. Additional ocular conditions recorded comprised strabismus subtypes including esotropia and exotropia, nystagmus, nasolacrimal duct obstruction, lens opacities or cataracts, glaucoma, and keratoconus. Ocular abnormalities were found in 58.0% to 97.2% of children with DS, with higher prevalence rates among Asian and European populations compared to American and African cohorts; though this variation may reflect differences in classified methodologies rather than true ethnic distinctions.

**Table 4:** Comparison of ophthalmic abnormality prevalence in children with Down syndrome.

	Country/ Region	Age	Sample size	Overall	Myopia	Hyperopia	Astigmatism	Visually significant refractive error	Strabismus	ET†	XT‡	Nystagmus	NLDO§	Cataract/ lens opacity	Glaucoma	Keratoconus
	**Asia**															
Current study, 2025	**Taiwan**	**0y-18y**	**148 (M:82, F:66)**	**89.2%**	**20.9%**	**55.4%**	**70.3%**	**75.7%**	**41.9%**	**34.5%**	**4.7%**	**27.7%**	**8.1%**	**10.1%**	**2.7%**	**0.0%**
Wong and Ho, 1997	Hong Kong	3m-13y	140 (M:82, F:58)	69.0%	12.0%	42.0%	8.0%	58.0%	22.0%	20.0%	1.8%	11.0%		4.0%	0.7%	0.0%
Merrick and Koslowe, 2001	Israel	5y-18y	86 (M:43, F;43)					91.0%	33.0%			9.0%		37.0%		13.0%
Kim et al., 2002	South Korea	6m-14y	123 (M:81, F:42)	91.0%	25.0%	28.0%	31.0%		25.0%	14.5%	10.5%	22.0%	17.0%	3.0%	0.8%	
Liza-Sharmini et al., 2006	Malaysia	1m-17y	60 (M:26, F:34)		29.2%	25.0%	8.3%		26.7%	26.7%	0.0%	33.3%	3.3%	13.3%	6.7%	0.0%
Kim et al., 2009	South Korea	N/A	172 (M:85, F:87)		40.1%	46.5%	66.8%		34.3%	22.1%	10.5%	29.1%				
Paudel et al., 2010	Nepal	4m-18y	36 (M:19, F:17)	97.2%	25.0%	55.0%	44.0%	80.0%	5.6%	0.0%	0.0%	28.0%	22.0%	5.0%		
Han et al., 2012	South Korea	2y-36y	41		48.8%	36.6%	51.2%		43.9%	23.4%	19.5%	31.7%				
Makateb et al., 2020	Iran	10y-30y	226 (M;118, F:108)		33.6%	45.6%	20.3%		23.4%	21.2%	0.9%	11.7%	2.9%			
	**Europe**															
Berk et al., 1996	Turkey	2m-25y	55 (M:38, F:17)		12.6%	52.7%	43.6%	87.3%	21.8%			12.7%	21.8%	20.0%		
Fimiani et al., 2007	Italy	1m-18y	157 (M:70, F:87)	100.0%	9.0%	59.0%	28.0%		36.0%	28.9%	7.1%	6.0%	22.0%	11.0%	0.0%	0.0%
Creavin et al., 2010	UK	0-16y	98	96.0%	17.0%	83.0%	74.0%		65.0%	50.1%	15.0%	9.0%		4.0%		
Ljubic et al. 2011	Macedonia and Croatia	1y-34y	170 (M:98, F:72)		20.7%	55.2%	72.4%		26.5%	18.8%	5.3%	11%				
Ljubic et al., 2015	Croatia	mean 10.6 y	51 (M:22, F:29)		20.4%	53.1%	68.8%		29.4%			2.0%		8.0%		
Purpura et al., 2019	Italy	2m-18m	42 (M;20, F:22)		11.9%	83.3%	45.2%	100.0%	21.4%			16.7%	7.1%	7.1%		
Ugurlu et al., 2020	Turkey	7y-18y	44 (M: 21, F:23)		29.5%	70.5%	70.5%		22.7%	18.2%	4.5%	2.3%		31.8%		
	**Americas**															
Caputo et al., 1989	USA	3.5m-26y	187 (M;102, F:85)		22.5%	21.0%	22.5%	30.0%	57.0%	51.0%	2.0%	29.0%	5.0%	11.2%	5.3%	
Roizen et al., 1994	USA	N/A	77	60.0%	14.3%	6.5%	6.5%	35.0%	27.0%	16.9%	1.4%	20.0%		5.0%	1.4%	0.0%
Umfress et al., 2019	USA	<18 y	689	61.0%			12.5%	38.0%	36.0%	29.6%	4.0%	17.0%	8.0%	6.2%		
	**Africa**															
Adio et al., 2012	Nigeria	6m-28y	42 (M:22, F:20)		38.1%	9.5%	28.6%	76.2%	9.5%	7.1%	2.4%	4.8%	2.4%			
Afifi et al., 2013	Egypt	2m-10y	90 (M:47, F:43)	58.0%				41.0%	14.0%			3.0%		6.0%	0.0%	0.0%
Aghaji et al., 2013	Nigeria	5-15y	30					76.40%	33.3%			13.3%		3.30%		

† ET: esotropia; ‡XT: exotropia; §NLDO: nasolacrimal duct obstruction; N/A: not available.

Refractive errors were the predominant finding in our cohort, consistent with published literature. Geographic variations were notable, with American studies [5,6,22] reporting lower prevalence rates (<40%). Our cohort showed myopia in 20.9%, hyperopia in 55.4%, and markedly higher astigmatism at 70.3%, aligning with Akinci et al’s [34] observation of lower myopia but higher hyperopia and astigmatism in DS children. The high prevalence of astigmatism in DS children can be attributed to the characteristic craniofacial features. The mechanical effect of upward slanting palpebral fissures common in Asian individuals [35] may explain the high astigmatism prevalence in our cohort. Furthermore, evidence suggests that the prevalence of substantial astigmatism in DS children tends to increase with age.

Analysis of refractive evolution in our cohort revealed that children with DS demonstrated an annual refractive shift of -0.16 ± 0.65 D in this Taiwanese population. Given the paucity of longitudinal refractive data in DS populations, we contextualized our findings through comparison with typically developing children. In non-DS urban children, documented annual myopic progression rates were -0.55 D in European and -0.82 D in Asian populations, with progression rates inversely correlating with baseline age [36]. While prolonged near-work activities have been strongly associated with myopia development in typically developing children [37], such sustained near-vision tasks are less frequently observed in children with DS, potentially contributing to their distinct refractive progression pattern.

Linear regression analysis identified age and baseline spherical equivalence as the critical determinants of myopic shifts in DS children. While typically developing children show prominent myopic shifts at younger ages due to axial length elongation [38], DS children demonstrate an altered pattern. Previous studies suggest impaired emmetropization in DS [8,9,35,39], where initial refractive errors tend to persist with age. However, older hyperopic DS children still exhibited pronounced myopic shifts with age [39], potentially due to the increased near-vision demands.

In myopic eyes, visually significant astigmatism emerged as the sole factor influencing myopic progression, consistent with findings in Chinese preschool children where greater astigmatism correlated with increased axial length elongation and myopic shift [40]. In hyperopic DS children, myopic shifts strongly correlated with male sex, age, esotropia, and baseline spherical equivalence. While meta-analysis data showed hyperopia inversely correlates with age in typically developing children [41], the relationship between hyperopia and strabismus in DS remains complex. Ljubic et al [24] reported lower strabismus rates in high-grade versus low-grade hyperopia, and Cho et al [42] found accommodative esotropia typically develops between ages two and three. However, still some studies found no significant association between strabismus and refractive errors in DS children [39].

Several limitations warrant consideration in our study. First, the challenges in accessing healthcare services for subjects with DS may result in selection bias, as parents of DS children with mild symptoms might be less likely to seek medical attention, potentially affecting the reported prevalence rates. Second, the inherent difficulties these children face during eye examinations challenged the acquisition of accurate refractive data. Third, high loss to follow-up rates resulted in a limited sample size.

In this comprehensive analysis of ophthalmic disorders in Taiwanese children with DS aged under 18 years, combined with an extensive literature review, we observed a high prevalence of visually significant refractive errors (75.7%), with astigmatism (70.3%) notably exceeding rates reported in previous studies of Asian children with DS. Our longitudinal analysis revealed a modest annual refractive change of −0.16 ± 0.65 diopters, with age and baseline spherical equivalence emerging as significant contributing factors, which was the first annual refractive changes statistics of Asian children with DS.

Future longitudinal studies with larger cohorts are needed to validate our findings on ophthalmic disorders in Asian children with DS. Our unprecedented data on annual refractive progression and the influence of demographic and ophthalmological factors contribute to the growing understanding of DS-related eye disorders in Asian populations. These insights may guide the development of targeted screening protocols and intervention strategies to address the unique ophthalmic needs of children with DS. Standardized classification methods and comprehensive follow-up protocols would further enhance our understanding of refractive evolution in this population.

## Supporting information

S1 Table Definitions of visually significant refractive error. (DOCX)

S1 Dataset DS raw data. (XLSX)

## References

[1] Grimm J, Heckl D, Klusmann J-H. Molecular mechanisms of the genetic predisposition to acute megakaryoblastic leukemia in infants with Down syndrome. Front Oncol. 2021;11:636633. doi: 10.3389/fonc.2021.636633 33777792 PMC7992977

[2] Lin S-Y, Hsieh C-J, Chen Y-L, Shaw SWS, Lin M-W, Chen P-C, et al. The impact of Down syndrome screening on Taiwanese Down syndrome births: a nationwide retrospective study and a screening result from a single medical centre. PLoS One. 2013;8(9):e75428. doi: 10.1371/journal.pone.0075428 24147155 PMC3798710

[3] Antonarakis SE, Skotko BG, Rafii MS, Strydom A, Pape SE, Bianchi DW, et al. Down syndrome. Nat Rev Dis Primers. 2020;6(1):9. doi: 10.1038/s41572-019-0143-7 32029743 PMC8428796

[4] Bull MJ, Trotter T, Santoro SL, Christensen C, Grout RW, Council on Genetics, et al. Health supervision for children and adolescents with Down syndrome. Pediatrics. 2022;149(5):e2022057010. doi: 10.1542/peds.2022-057010 35490285

[5] Roizen NJ, Mets MB, Blondis TA. Ophthalmic disorders in children with Down syndrome. Dev Med Child Neurol. 1994;36(7):594–600. 8034121 10.1111/j.1469-8749.1994.tb11896.x

[6] Caputo A, Wagner R, Reynolds D, Guo S, Goel A. Down syndrome clinical review of ocular features. Clin Pediatr (Phila). 1989;28(8):355–8. 2527102 10.1177/000992288902800804

[7] Jaeger EA. Ocular findings in Down’s syndrome. Trans Am Ophthalmol Soc. 1980;78:808–45. 6455003 PMC1312160

[8] Watt T, Robertson K, Jacobs RJ. Refractive error, binocular vision and accommodation of children with Down syndrome. Clin Exp Optom. 2015;98(1):3–11. doi: 10.1111/cxo.12232 25395109

[9] Afifi HH, Abdel Azeem AA, El-Bassyouni HT, Gheith ME, Rizk A, Bateman JB. Distinct ocular expression in infants and children with Down syndrome in Cairo, Egypt: myopia and heart disease. JAMA Ophthalmol. 2013;131(8):1057–66. doi: 10.1001/jamaophthalmol.2013.644 23764677

[10] Ljubic A, Trajkovski V, Stankovic B. Strabismus, refractive errors and nystagmus in children and young adults with Down syndrome. Ophthalmic Genet. 2011;32(4):204–11. doi: 10.3109/13816810.2011.592175 21728809

[11] Paudel N, Leat SJ, Adhikari P, Woodhouse JM, Shrestha JB. Visual defects in Nepalese children with Down syndrome. Clin Exp Optom. 2010;93(2):83–90. doi: 10.1111/j.1444-0938.2010.00458.x 20406257

[12] Liza-Sharmini AT, Azlan ZN, Zilfalil BA. Ocular findings in Malaysian children with Down syndrome. Singapore Med J. 2006;47(1):14–9. 16397715

[13] Creavin AL, Brown RD. Ophthalmic assessment of children with down syndrome: is England doing its bit?. Strabismus. 2010;18(4):142–5. doi: 10.3109/09273972.2010.529232 21091335

[14] de Weger C, Boonstra N, Goossens J. Bifocals reduce strabismus in children with Down syndrome: evidence from a randomized controlled trial. Acta Ophthalmol. 2020;98(1):89–97. doi: 10.1111/aos.14186 31313886 PMC7003890

[15] da Cunha RP, Moreira JB. Ocular findings in Down’s syndrome. Am J Ophthalmol. 1996;122(2):236–44. doi: 10.1016/s0002-9394(14)72015-x 8694092

[16] Wallace DK, Morse CL, Melia M, Sprunger DT, Repka MX, Lee KA, et al. Pediatric eye evaluations preferred practice pattern®: I. Vision screening in the primary care and community setting; II. Comprehensive ophthalmic examination. Ophthalmology. 2018;125(1):P184–227. doi: 10.1016/j.ophtha.2017.09.032 29108745

[17] Donahue SP, Arthur B, Neely DE, Arnold RW, Silbert D, Ruben JB, et al. Guidelines for automated preschool vision screening: a 10-year, evidence-based update. J AAPOS. 2013;17(1):4–8. doi: 10.1016/j.jaapos.2012.09.012 23360915

[18] Fong AHC, Shum J, Ng ALK, Li KKW, McGhee S, Wong D. Prevalence of ocular abnormalities in adults with Down syndrome in Hong Kong. Br J Ophthalmol. 2013;97(4):423–8. doi: 10.1136/bjophthalmol-2012-302327 23376568

[19] Little J-A, Mahil A-DS, Richardson P, Woodhouse JM, Vinuela-Navarro V, Saunders KJ. In-vivo anterior segment OCT imaging provides unique insight into cerulean blue-dot opacities and cataracts in Down syndrome. Sci Rep. 2020;10(1):10031. doi: 10.1038/s41598-020-66642-1 32572106 PMC7308272

[20] Ugurlu A, Altinkurt E. Ophthalmologic manifestations and retinal findings in children with Down syndrome. J Ophthalmol. 2020;2020:9726261. doi: 10.1155/2020/9726261 32089873 PMC7029299

[21] Makateb A, Hashemi H, Farahi A, Mehravaran S, Khabazkhoob M, Asgari S. Ocular alignment, media, and eyelid disorders in Down syndrome. Strabismus. 2020;28(1):42–8. 31830843 10.1080/09273972.2019.1699582

[22] Umfress AC, Hair CD, Donahue SP. Prevalence of ocular pathology on initial screening and incidence of new findings on follow-up examinations in children with trisomy 21. Am J Ophthalmol. 2019;207:373–7. doi: 10.1016/j.ajo.2019.06.006 31220432

[23] Purpura G, Bacci GM, Bargagna S, Cioni G, Caputo R, Tinelli F. Visual assessment in Down Syndrome: the relevance of early visual functions. Early Hum Dev. 2019;131:21–8. doi: 10.1016/j.earlhumdev.2019.01.020 30818135

[24] Ljubic A, Trajkovski V, Tesic M, Tojtovska B, Stankovic B. Ophthalmic manifestations in children and young adults with Down syndrome and congenital heart defects. Ophthalmic Epidemiol. 2015;22(2):123–9. doi: 10.3109/09286586.2015.1017652 25777312

[25] Aghaji AE, Lawrence L, Ezegwui I, Onwasigwe E, Okoye O, Ebigbo P. Unmet visual needs of children with Down syndrome in an African population: implications for visual and cognitive development. Eur J Ophthalmol. 2013;23(3):394–8. doi: 10.5301/ejo.5000222 23335310

[26] Han DH, Kim KH, Paik HJ. Refractive errors and strabismus in Down’s syndrome in Korea. Korean J Ophthalmol. 2012;26(6):451–4. doi: 10.3341/kjo.2012.26.6.451 23204801 PMC3506820

[27] Adio AO, Wajuihian SO. Ophthalmic manifestations of children with Down syndrome in Port Harcourt, Nigeria. Clin Ophthalmol. 2012;6:1859–64. doi: 10.2147/OPTH.S36685 23185113 PMC3501839

[28] Kim U, Hwang J-M. Refractive errors and strabismus in Asian patients with Down syndrome. Eye (Lond). 2009;23(7):1560–4. doi: 10.1038/eye.2008.309 18849915

[29] Fimiani F, Iovine A, Carelli R, Pansini M, Sebastio G, Magli A. Incidence of ocular pathologies in Italian children with Down syndrome. Eur J Ophthalmol. 2007;17(5):817–22. doi: 10.1177/112067210701700521 17932861

[30] Kim JH, Hwang J-M, Kim HJ, Yu YS. Characteristic ocular findings in Asian children with Down syndrome. Eye (Lond). 2002;16(6):710–4. doi: 10.1038/sj.eye.6700208 12439664

[31] Merrick J, Koslowe K. Refractive errors and visual anomalies in Down syndrome. Downs Syndr Res Pract. 2001;6(3):131–3. 11501216 10.3104/reports.105

[32] Wong V, Ho D. Ocular abnormalities in Down syndrome: an analysis of 140 Chinese children. Pediatr Neurol. 1997;16(4):311–4. doi: 10.1016/s0887-8994(97)00029-5 9258964

[33] Berk AT, Saatci AO, Erçal MD, Tunç M, Ergin M. Ocular findings in 55 patients with Down’s syndrome. Ophthalmic Genet. 1996;17(1):15–9. doi: 10.3109/13816819609057864 8740693

[34] Akinci A, Oner O, Bozkurt OH, Guven A, Degerliyurt A, Munir K. Refractive errors and strabismus in children with Ddown syndrome: a controlled study. J Pediatr Ophthalmol Strabismus. 2009;46(2):83–6. doi: 10.3928/01913913-20090301-04 19343969 PMC4282924

[35] Haugen OH, Høvding G, Lundström I. Refractive development in children with Down’s syndrome: a population based, longitudinal study. Br J Ophthalmol. 2001;85(6):714–9. doi: 10.1136/bjo.85.6.714 11371494 PMC1723994

[36] Donovan L, Sankaridurg P, Ho A, Naduvilath T, Smith E 3rd, Holden B. Myopia progression rates in urban children wearing single-vision spectacles. Optom Vis Sci. 2012;89(1):27–32. 21983120 10.1097/OPX.0b013e3182357f79 PMC3249020

[37] Tsai T-H, Liu Y-L, Ma I-H, Su C-C, Lin C-W, Lin LL-K, et al. Evolution of the prevalence of myopia among Taiwanese school children: a review of survey data from 1983 through 2017. Ophthalmology. 2021;128(2):290–301. doi: 10.1016/j.ophtha.2020.07.017 32679159

[38] Chen Z, Gu D, Wang B, Kang P, Watt K, Yang Z. Significant myopic shift over time: sixteen-year trends in overall refraction and age of myopia onset among Chinese children, with a focus on ages 4–6 years. J Glob Health. 2023;13:04144. 37934967 10.7189/jogh.13.04144 PMC10630697

[39] Cregg M, Woodhouse JM, Stewart RE, Pakeman VH, Bromham NR, Gunter HL, et al. Development of refractive error and strabismus in children with Down syndrome. Invest Ophthalmol Vis Sci. 2003;44(3):1023–30. doi: 10.1167/iovs.01-0131 12601024

[40] Fan DSP, Rao SK, Cheung EYY, Islam M, Chew S, Lam DSC. Astigmatism in Chinese preschool children: prevalence, change, and effect on refractive development. Br J Ophthalmol. 2004;88(7):938–41. doi: 10.1136/bjo.2003.030338 15205242 PMC1772230

[41] Castagno VD, Fassa AG, Carret MLV, Vilela MAP, Meucci RD. Hyperopia: a meta-analysis of prevalence and a review of associated factors among school-aged children. BMC Ophthalmol. 2014;14:163. doi: 10.1186/1471-2415-14-163 25539893 PMC4391667

[42] Cho YA, Ryu WY. Changes in refractive error in patients with accommodative esotropia after being weaned from hyperopic correction. Br J Ophthalmol. 2015;99(5):680–4. doi: 10.1136/bjophthalmol-2014-305991 25416183

