# Supplementary material for: Prevalence of ophthalmic abnormalities and refractive changes in Taiwanese children with Down syndrome
Source: PLoS One. 2025 May 21;20(5):e0324366. doi: 10.1371/journal.pone.0324366 (PMC12094766; doi:10.1371/journal.pone.0324366)
Supplement: S1 Table — (DOCX) [file pone.0324366.s001.docx]

**Supplementary table 1. Definitions of visually significant refractive errors**

|  | Refractive error in diopters | | | | |
| --- | --- | --- | --- | --- | --- |
| Ametropia | Age < 1 y/o | Age 1-2 y/o | Age 2-3 y/o | Age 3-4 y/o | Age > 4 y/o |
| Isometropia |  | | | | |
| Myopia | > 5.0D | > 4.0D | > 3.0D | > 2.5D | > 1.5D |
| Hyperopia without deviation | > 6.0D | > 5.0D | > 4.5D | > 3.5D | > 3.5D |
| Hyperopia with esotropia | > 2.0D | > 2.0D | > 2.0D | > 1.5D | > 1.5D |
| Astigmatism | > 3.0D | > 2.5D | > 2.0D | > 1.5D | > 1.5D |
| Anisometropia |  | | | | |
| Myopia | > 4.0D | > 3.0D | > 3.0D | > 2.5D | > 1.5D |
| Hyperopia | > 2.5D | > 2.0D | > 1.5D | > 1.5D | > 1.5D |
| Astigmatism | > 2.5D | > 2.0D | > 2.0D | > 1.5D | > 1.5D |

The decision of visually significant refractive errors was made in accordance with the definitions in the 2018 edition of the *Pediatric Eye Evaluations Preferred Practice Pattern* published by the American Academy of Ophthalmology (AAO) and the 2013 version of American Association for Pediatric Ophthalmology and Strabismus (AAPOS) *Vision Screening Committee Guidelines*.
